# Enhanced behavioural and neural sensitivity to punishments in chronic pain and fatigue

**DOI:** 10.1093/brain/awae408

**Published:** 2024-12-30

**Authors:** Flavia Mancini, Pranav Mahajan, Anna á V Guttesen, Jakub Onysk, Ingrid Scholtes, Nicholas Shenker, Michael Lee, Ben Seymour

**Affiliations:** Department of Engineering, Computational and Biological Learning Unit, University of Cambridge, Cambridge CB2 1PZ, UK; Nuffield Department of Clinical Neurosciences, Wellcome Centre for Integrative Neuroimaging, FMRIB, University of Oxford, Oxford OX3 9DU, UK; Nuffield Department of Clinical Neurosciences, Wellcome Centre for Integrative Neuroimaging, FMRIB, University of Oxford, Oxford OX3 9DU, UK; Department of Engineering, Computational and Biological Learning Unit, University of Cambridge, Cambridge CB2 1PZ, UK; Queen Square Institute of Neurology and Mental Health Neuroscience Department, Division of Psychiatry, Applied Computational Psychiatry Lab, Max Planck Centre for Computational Psychiatry and Ageing Research, University College London, London WC1B 5EH, UK; Rheumatology Research Unit, Cambridge University Hospital, Cambridge CB2 0QQ, UK; Department of Medicine, University of Cambridge, Cambridge CB2 0QQ, UK; Rheumatology Research Unit, Cambridge University Hospital, Cambridge CB2 0QQ, UK; Department of Medicine, University of Cambridge, Cambridge CB2 0QQ, UK; Department of Medicine, University of Cambridge, Cambridge CB2 0QQ, UK; Nuffield Department of Clinical Neurosciences, Wellcome Centre for Integrative Neuroimaging, FMRIB, University of Oxford, Oxford OX3 9DU, UK; Institute of Biomedical Engineering, University of Oxford, Oxford OX3 7DQ, UK

**Keywords:** reinforcement learning, chronic pain, fatigue, arthritis, insula, decision-making

## Abstract

Chronic pain and fatigue in musculoskeletal disease contribute significantly to disability, and recent studies suggest an association with reduced motivation and excessive fear avoidance. In this behavioural neuroimaging study, we aimed to identify the specific behavioural and neural changes associated with musculoskeletal pain and fatigue during reward and loss decision-making.

Twenty-nine participants with chronic inflammatory arthritis and 28 healthy controls performed an instrumental learning task (four-armed bandit) during 3 T brain functional MRI. Computational analyses with reinforcement learning models were used to quantify the hidden variables involved in reward and loss decision-making, compare them across groups and, finally, relate them to brain activity.

We found that participants with chronic pain had higher sensitivity to punishments and increased activity associated with the punishment prediction error in the right posterior insular cortex, putamen, pallidum and dorsolateral prefrontal cortex. Functional network connectivity analysis showed that insula centrality was correlated with subjective reports of fatigue and pain during the task.

The findings of this exploratory study suggest that pain and fatigue in chronic pain relate to objective behavioural changes in loss decision-making, which can be mapped to a specific pattern of activity in brain circuits of motivation and decision-making. The proposed parametric signature, characterized most notably by increased punishment sensitivity, is distinct from patterns previously reported in psychiatric conditions and it aligns with predictions of the fear avoidance model of pain.

## Teaser

In a behavioural neuroimaging study, Mancini *et al.* demonstrate that people with chronic inflammatory arthritis show increased sensitivity to punishments when making decisions about volatile wins and losses. This behavioural change can be mapped to a specific pattern of activity in neural networks involved in motivation.

## Introduction

Chronic pain and fatigue are the cardinal symptoms of musculoskeletal disease, and they lead to a level of disability that has significant socioeconomic cost.^1^ Although multiple factors can contribute to the risk of developing chronic pain, a key hypothesis is that reduced central motivational drive to engage in physical activity leads to physical deconditioning that, in turn, exacerbates pain, a corollary of the fact that physiotherapy is clearly beneficial in most subacute and chronic musculoskeletal conditions.^2,3^ This ‘fear avoidance’ hypothesis links with other neurobiological and cognitive factors known to contribute to risk for pain chronification, embedded within the broader biopsychosocial model that captures the complexity of chronic pain in everyday clinical contexts.^4^ The mechanism by which pain reduces central motivation therefore represents a critical link in the pathway to chronic pain and fatigue development,^5^ but its nature and neurobiology are not fully understood.

Injuries are common across animal species (e.g. after accidents and within/cross-species contests) and lead to recognizable protective and recuperative behaviours.^6^ These are presumed to be evolutionarily adaptive because the injured state makes animals more vulnerable to further harm, hence being extra careful whilst recovering benefits survival.^7^ Peripheral and spinal sensitization to pain provide one route to hyper-protective behaviour, and excessive pain hypersensitivity is a key focus of many current models of chronic pain. But recent ecological studies indicate a broader hypersensitivity to threat that goes beyond pain, suggesting a central, supramodal enhanced sensitivity to punishments as a mechanism of behavioural homeostasis after an injury, and a potential contributing factor to chronic pain and disability.^8^ Although enhanced punishment sensitivity would be adaptive for simple musculoskeletal injuries encountered in ecological contexts, it would ultimately be maladaptive in the context of clinical musculoskeletal conditions, where inactivity is detrimental.^5^

Computational models of the neural circuits of learning and motivation allow us to dissect its core information-processing steps, thus helping to identify the underlying components of post-injury homeostasis, such as modulation of punishment sensitivity.^9^ A similar approach has been applied to human behavioural experiments to identify, for example, reduced reward sensitivity in major depressive disorder and aversive generalization in anxiety disorder.^10-12^ In this study, we aimed to identify: (i) whether there is a specific behavioural signature of pain and fatigue manifest during reward and loss decision-making; and (ii) if there is, whether it relates to specific, localizable activity in the brain, particularly in circuits associated with motivation and learning.

**Figure 1:**
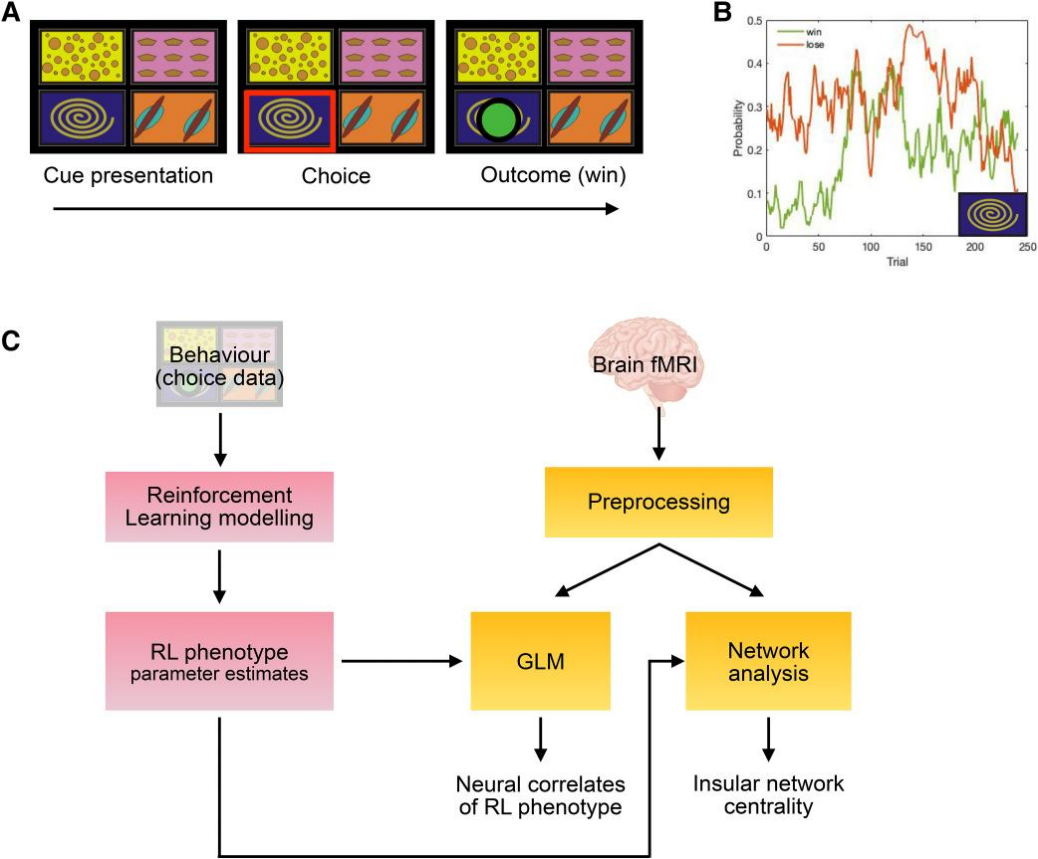
**Task design and analysis workflow.** (**A**) At the beginning of each trial, four cues were presented, and participants chose one of them. Each cue was associated with an independent probability of winning and losing a token. After each choice, the outcome was displayed (green coin = win £1). (**B**) Example of the evolution of the probability of winning (green line) and losing a coin (orange line) associated with the chosen option. (**C**) Outline of the analysis pipeline. Five variants of reinforcement learning (RL) models were used to fit behavioural choice data, and their fit was compared to identify the best-fitting model. The parameter estimates of arthritis participants and controls were then compared using Bayesian statistics. Parameters from the winning models were then related to blood oxygenation level-dependent activity as regressors of interest in general linear models (GLM) and network analyses focused on insular subregions.

Reward and loss decision-making captures a fundamental process that underlies much of our everyday behaviour: doing things that benefit us and avoiding things that do not. We studied this using a four-armed bandit task^10,13,14^ (Fig. 1) in 30 patients with painful inflammatory arthritis and in 30 age- and sex-matched controls. In the four-armed bandit task, participants were presented with four cues, and they needed to choose one of them in each trial. Each cue was associated with an independent, non-stationary probability of winning or losing a coin worth £1. Thus, participants needed to learn constantly and independently about rewards and losses, allowing us to dissociate differences in reward and loss learning. Computational modelling of behavioural data was then used to interrogate brain activity to find the corresponding neural processing steps and their relationship to pain and fatigue. In the human brain, the mesolimbic and insula regions are thought to play a key role in pain-related motivation and avoidance learning,^15-18^ alongside behavioural homeostasis (e.g. in illness and sickness states^19^). We reasoned that the insula, in particular, might link the physiological identification of injury with responsiveness to punishments, which might ultimately contribute to the pain and fatigue phenotype.

## Materials and methods

### Participants

Initially, we engaged 15 local patients with chronic pain to help us design the study and iterated preliminary versions of the protocol using a systems engineering approach. The study was approved by the central National Health Service Health Research Authority ethics committee (REC: 17/SW/0113, IRAS: 216259). After the design was finalized, we screened 80 volunteers and recruited 30 patients with chronic rheumatoid and psoriatic arthritis and 30 healthy age-matched controls. Patients were recruited at Rheumatology, Cambridge University Hospitals. All participants gave informed written consent according to the Helsinki Guidelines. Three participants did not complete the full study and were therefore excluded, leaving us with 57 participants in total (28 controls and 29 patients; 45 females). Patients were 53.83 ± 8.09 years old (range 36–66 years; 27 females), and controls were 51.50 ± 10.77 years old (range 33–69 years; 18 females).

Participants were screened using a non-standardized demographic questionnaire and the following standardized questionnaires: Beck Depression Inventory II, Brief Pain Inventory, McGill Pain Assessment, Functional Assessment of Chronic Illness Therapy Fatigue Scale, Short form PROMIS Sleep Scale. Trained staff carried out arthritis assessments with standardized joint assessment disease activity score, rheumatoid classification [American College of Rheumatology/European Alliance of Associations for Rheumatology (ACR/EULAR) criteria] and psoriatic classification. Patients with disease activity (disease activity score > 2.6) or subjectively high fatigue scores [>4 on scale from 0 = ‘no fatigue’ to 10 = ‘worst fatigue imaginable’, Functional Assessment of Chronic Illness Therapy/Fatigue Scale (FACIT) < 35] were included in the study. Controls had no clinically significant disease activity (disease activity score < 2.6) or subjective fatigue (<4 on a scale from 0 = no fatigue to 10 = worst fatigue imaginable, FACIT > 30). Participants with other explanations for fatigue (anaemia, unstable thyroid, heart conditions, diabetes, recent cancer treatment or recent surgery) and contra-indications for MRI safety were considered not eligible for the study. The demographic and clinical details of the participants who completed the study are reported in the Supplementary material, ‘Clinical and demographic data’ section and Supplementary Table 1.

### Procedure

Participants took part in an initial screening and training session and in a subsequent MRI session. The two sessions were separated by 5–73 days, depending on participant, site and scanner availability. Throughout both sessions and in between tasks/runs, participants were asked to rate their fatigue and pain on a scale of 0–10 (0 = no fatigue/pain, 10 = worst fatigue/pain imaginable).

#### Four-armed bandit task

Participants underwent a four-armed bandit decision-making task during MRI, following some practice. In each trial, participants could choose between four abstract boxes (i.e. cues) presented on the screen, and they needed to choose one of them in each trial (Fig. 1). Each cue was associated with an independent probability of winning or losing a coin worth £1. The mean reward and punishment rate were independent of each other and noisy, changing slowly across trials as a Gaussian random walk. After selecting one of the cues, their choice yielded one of four possible outcomes: one green token (win £1), one red token (lose £1), one green and one red token (win £1 but lose £1, effectively £0 when added at the end of the game) or no outcome. Thus, participants need to learn constantly and independently about rewards and losses, allowing us to dissociate differences in reward and loss learning. The sum of positive minus negative tokens was exchanged for money at the end of the experiment. The participants completed 240 trials over four runs.

#### Inflammatory markers

Blood samples were obtained from all participants during the second visit, and the following inflammatory markers were evaluated: erythrocyte sedimentation rate (ESR), IFNγ, IL-10, IL-12p70, IL-13, IL-1β, IL-2, IL-4, IL-6, IL-8, TNFα. General health was assessed in full blood count and liver function samples.

#### Actigraphy and daily ratings

Participants recorded daily ratings and wore an ActiGraph GT9X Link (Actigraph, LLC) on the wrist, 24 h a day, over the course of 5–7 days. The wearable device contains a piezo-electric sensor that generates a voltage when the device undergoes a change in acceleration (three-axial). It was set to record 60 s epochs. Participants removed the wristbands only for showering or swimming. During this week, participants rated their fatigue and pain (‘how much fatigue/pain do you feel right now on a scale of 0 to 10?’, whereby 0 = no fatigue/pain and 10 = worst fatigue/pain imaginable). The participants set their alarms in the morning (09:00/09:30) and evening (19:30/20:00) and noted their current rating down on a questionnaire that was previously provided by the experimenters. Participants also recorded their daily consumption of caffeine, alcohol and medication on a questionnaire. They noted how many units were consumed and the details of the product (e.g. filter coffee, paracetamol, wine). Analysis and results of actigraphy are reported in the Supplementary material.

### Modelling of choice behaviour

We used a hierarchical Bayesian inference approach to fit multiple variants of reinforcement learning models to the choice data, first to all pooled participants, then separately for the two groups. These are biological informed models, arising from basic animal learning theory, which are known to capture behaviour and brain activity during this type of task.^9^ The hierarchical Bayesian approach allows for individual differences while aggregating information across participants of the same group and make meaningful group comparisons.^20^

We simulated and fitted five reinforcement learning models based on non-probabilistic delta rules, whereby the learning rates are fixed and driven by discrepancies between the estimate of the expected value and observed values. In all models, we tracked the *Q*-value of the four cues as the sum of the *Q*-values for rewards ($Q^{r}$) and punishments ($Q^{p}$). We calculated the reward and punishment prediction errors as the difference between the expected and observed value (Equations 1 and 2):


(1) $$\begin{array}{c} \;\delta_{t(i)}^{p}=O_{t(i)}^{p}-Q_{t(i)}^{p} \end{array}$$

(2) $$\begin{array}{c} \;\delta_{t(i)}^{r}=O_{t(i)}^{r}-Q_{t(i)}^{r} \end{array}$$

In all models, the prediction error was scaled by a free sensitivity parameter (reward sensitivity *R* or punishment sensitivity *P*) (Equations 3 and 4), as in previous studies^10^:


(3) $$\begin{array}{c} \;\delta_{t(i)}^{p}=P\times (O_{t(i)}^{p}-Q_{t(i)}^{p}) \end{array}$$

(4) $$\begin{array}{c} \;\delta_{t(i)}^{r}=R\times (O_{t(i)}^{r}-Q_{t(i)}^{r}) \end{array}$$

The models differed also according to the update rule, and the presence of lapses and forgetting in choice behaviour. In all models, the learning rate was constant across trials. In Models 1 and 2, the learning rate for rewards was calculated as the sum of two free parameters, α+β, and the learning rate for punishments was calculated as the difference of these free parameters, α−β:


(5) $$\begin{array}{c} \;Q_{t(i)}^{r}=Q_{t(i)}^{r}+(\alpha +\beta )\times \delta_{t(i)}^{r} \end{array}$$

(6) $$\begin{array}{c} \;Q_{t(i)}^{p}=Q_{t(i)}^{p}+(\alpha -\beta )\times \delta_{t(i)}^{p} \end{array}$$

In Models 3–5, the learning rates for rewards and punishments were estimated as two independent free parameters, i.e. $\alpha^{r}$ and $\alpha^{p}$:


(7) $$\begin{array}{c} \;Q_{t(i)}^{r}=Q_{t(i)}^{r}+\alpha^{r}\times \delta_{t(i)}^{r} \end{array}$$

(8) $$\begin{array}{c} \;Q_{t(i)}^{p}=Q_{t(i)}^{p}+\alpha^{p}\times \delta_{t(i)}^{p} \end{array}$$

Models 1 and 3 additionally had a decay free parameter *d* to describe the rate of forgetting in the tracking of $Q^{r}$ and $Q^{p}$ (Equations 9 and 10), as in the study by Aylward *et al*.^10^:


(9) $$\begin{array}{c} \;Q_{t(i)}^{p}=(1-d)\times Q_{t-1(i)}^{p} \end{array}$$

(10) $$\begin{array}{c} \;Q_{t(i)}^{r}=(1-d)\times Q_{t-1(i)}^{r} \end{array}$$

All models used a softmax policy to determine choice probability P by combining the *Q*-values of rewards and punishments:


(11) $$\begin{array}{c} \;P_{t(i)}=\frac{exp(Q_{t(i)}^{r}+Q_{t(i)}^{p})}{\sum_{j}\;exp(Q_{t(j)}^{r}+Q_{t(j)}^{p})} \end{array}$$

The softmax policy in Models 1–4 included a free parameter *ξ* that characterized lapses in choice behaviour, in a similar manner to the study by Aylward *et al*.^10^:


(12) $$\begin{array}{c} \;P_{t(i)}=\frac{exp(Q_{t(i)}^{r}+Q_{t(i)}^{p})}{\sum_{j}\;exp(Q_{t(j)}^{r}+Q_{t(j)}^{p})}\times (1-\xi )+\frac{\xi }{4} \end{array}$$

Table 1 summarizes the free parameters of the different models we simulated and fitted to the data.

**Table:** Table 1 Summary of model parameters

Model no.	Sensitivity	Reward LR	Punishment LR	Decay	Lapses
1	R, P	*α* + *β*	*α* − *β*	d	*ξ*
2	R, P	*α* + *β*	*α* − *β*	n/a	*ξ*
3	R, P	*α^r^*	*α^p^*	d	*ξ*
4	R, P	*α^r^*	*α^p^*	n/a	*ξ*
5	R, P	*α^r^*	*α^p^*	n/a	n/a

Free parameters of the models that were fitted to the behavioural data. *α*, *β* = learning rate parameters; *α^p^* = punishment learning rate; *α^r^* = reward learning rate; d = decay; *ξ* = lapse; LR = learning rate; n/a = not applicable; P = punishment sensitivity; R = reward sensitivity.

We used hierarchical Bayesian methods (Hamiltonian Monte Carlo) to estimate model parameters, with the HBayesDM and Stan package for R^20,21^ in R. For each model, we fitted four chains, with 1000 burn-in samples and 2000 samples, to the following data: choice (1:4), win (0, 1) and loss (0, −1) for each trial. Parameter and model recovery analyses are reported in Supplementary Tables 2–5 and Supplementary Fig. 2.

Parameters for all models were fitted separately for each group and for all participants pooled in a common group. The winning model was defined as the model with the lowest leave-one-out information criterion (LOOIC). We also estimated the difference in expected predictive accuracy of models through the difference in expected log point-wise predictive density (ELPD).^22^ By taking the ratio between the ELPD difference and the standard error (SE) of the difference, we obtained the sigma effect, which is a heuristic for significance of such model differences.

We compared parameter estimates from the winning model across the two groups using 95% high density intervals (HDI). For each comparison, we calculated the difference in the hyper-parameters between groups and reported the 95% HDI of the difference. In the Bayesian scenario, a significant group difference is indicated by the interval not containing the value zero.

### MRI data acquisition

First, we collected a T_1_-weighted magnetization-prepared rapid gradient-echo structural scan (voxel size 1 mm isotropic) on a 3 T Siemens Magnetom Prisma scanner (Siemens Healthcare), equipped with a 64-channel head coil, at the Wolfson Brain Imaging Centre, Cambridge. Then we collected a resting state scan of 240 volumes and four task functional MRI (fMRI) sessions of 264 volumes using the same multiband echo planar imaging (EPI) sequence (repetition time = 2000ms, echo time= 30 ms, flip angle = 82°, slices per volume = 64, multiband acceleration factor 2, interleaved slice mode, Grappa 2, voxel size 2 mm isotropic, A > P phase-encoding; this included four dummy volumes, in addition to those pre-discarded by the scanner). To correct for inhomogeneities in the static magnetic field, we imaged four volumes using an EPI sequence identical to that used in task fMRI, inverted in the posterior-to-anterior phase encoding direction.

### MRI data analyses

#### Preprocessing

The anatomical images were reoriented to standard orientation, automatically cropped and bias-field corrected using ‘fsl_anat’ (FMRIB’s Software Library, www.fmrib.ox.ac.uk/fsl). The brain was extracted with the ANTs tool ‘antsBrainExtraction’ (https://github.com/ANTsX/ANTs). Functional images had the first four volumes removed, were motion corrected using the FSL tool ‘mcflirt’, three-dimensionally time shifted using the AFNI program ‘3dTshift’ and a Fourier method (https://afni.nimh.nih.gov/). Fieldmap correction was applied using HySCO, Hyperelastic Susceptibility Artefact Correction.^23^ Non-brain data were removed using the FSL tool BET. Spatial smoothing was applied using a Gaussian kernel of full-width at half-maximum of 8.0 mm. The entire four-dimensional dataset was grand-mean intensity normalized by a single multiplicative factor, and high-pass temporal filtering was applied (Gaussian-weighted least-squares straight line fitting, with sigma = 50.0 s) using FSL.

#### Generalized linear modelling

First- and second-level generalized linear modelling analyses were conducted using FEAT (FMRI Expert Analysis Tool) v.6.00, part of FSL. All univariate imaging results were obtained from a single generalized linear model. We investigated neural correlates using Model 1. All model predictors were generated with the group mean fitted parameters to minimize noise. First-level regressors included onset times and values for the reward outcomes, punishment outcomes, reward prediction error, punishment prediction error (modelled at the time of the outcome) and regressors of no interest, such as the cue onset and keypress time. Sessions within subject were not concatenated but modelled separately, then averaged using a mixed-effect model (second-level analysis). Third-level analyses were two-group difference models, in which we compared the positive and negative means of reward or punishment prediction errors between the arthritis and control groups; as covariates, we included the sensitivity parameter (a constant) and age, de-meaned across all subjects. Finally, we applied a cluster threshold *z >* 2.6, *P <* 0.05, and we report only clusters that survived this multiple-comparison correction.

#### Connectivity analyses

Filtered and preprocessed functional images were registered to standard format (MNI template), then parcellated into 180 regions of interest using the Glasser functional atlas.^24^ Each region of interest combines the symmetric region in the brain, i.e. right and left regions jointly form one parcel. The time series for each parcel were extracted as the mean of the time series corresponding to all voxels in that parcel. This process was performed for all four runs of task-fMRI and one run of rest-fMRI, and we performed elementwise summation of the four task-fMRI time series runs, making it comparable with the rest-fMRI.

Fully connected networks were constructed, with edge strength between two nodes being the Pearson correlation between the time series corresponding to the nodes. We then thresholded the Pearson’s full correlation matrices to a produce binary adjacency matrix (consisting of ones and zeros) for each participant, as done in our previous studies.^25^ Each of the correlation matrices was thresholded in an adaptive manner to produce an adjacency matrix with a 10% link density. This value was chosen based on previous network topology studies that have found such a link density to provide optimal discriminative ability.^25-29^ We then computed the following nodal centrality measures for parcels corresponding to insular subregions: degree centrality, eigenvector centrality, betweenness centrality, closeness centrality and load centrality.

Glasser’s atlas, being a functional atlas, has several parcels, some of which may overlap with the anatomical insular region. We found the Glasser parcels that correspond to the anatomical insula region by cross-checking the regions that intersect/overlap with the left and right insula regions in the Automated Anatomical Labelling atlas, in FSLeyes. This resulted in 10 parcels of the Glasser atlas. Then, we performed Bayesian Pearson correlation of insular nodal centrality metrics with clinical scores and model parameters, only for the patient group. We did not include any healthy subjects because they have normal clinical scores and no pain, and correlations would have risked being confounded by group differences.

We correlated the nodal centrality metrics of these insular subregions with several scores and model variables: daily average pain, daily average fatigue, daily average sleep quality, disease activity score/DAPSA score, BDI score, GAD score, BPI severity, BPI interference, McGill questionnaire score, FACIT score, PROMIS (raw score), immunological variables (ESR, IFNγ, IL-10, IL-12p70, IL-13, IL-1β, IL-2, IL-4, IL-6, IL-8 and TNFα) and model parameters.

We controlled for multiple comparisons using a Bayesian false discovery rate (FDR) approach based on posterior probabilities derived from Bayes’ theorem. This approach follows the Bayesian interpretation of the FDR, as described by Storey.^30^ First, we computed the log Bayes factors (logBF10) for each test and transformed them into posterior probabilities $P(H_{1}|\mathrm{data})$ using Bayes’ rule with equal priors [$P(H_{0})=P(H_{1})=0.5$]:


(13) $$P(H_{1}|\mathrm{data})=\frac{\mathrm{BF}10}{1+\mathrm{BF}10}.$$

The posterior probabilities were then ranked in descending order, and the posterior error probabilities (PEPs) were computed as:


(14) $$\mathrm{PEP}=1-P(H_{1}|\mathrm{data}).$$

The *Q*-values were calculated as the cumulative average of the ranked PEPs, i.e. for the *i*th test:


(15) $$q_{i}=\frac{1}{i}\sum_{\;j=1}^{i}\mathrm{PE}P_{j}.$$

Finally, we applied a cut-off to the *Q*-values corresponding to the desired Bayesian FDR threshold, retaining only those tests with *Q*-values below the threshold.

For this study, we applied a conservative FDR threshold of α=0.01, to control for the rate of false positives (type I errors) in multiple testing.

## Results

### Choice behaviour

Basic performance measures (net gains, reaction times and frequency of choice switches) were comparable between patients and controls (see the Supplementary material, ‘Additional task results’ and Supplementary Fig. 1). This was expected; given that the task is effectively a dynamic learning environment for probabilistic outcomes, different levels of performance do not yield big differences in outcomes, which protects against the potentially confounding effects of accruing different winnings. In contrast, any differences between groups are embedded in the trial-by-trial dynamics because of exploration and learning.

To capture these learning effects, we used a hierarchical Bayesian inference approach to fit multiple variants of reinforcement learning models to the choice data. The behavioural data of the pooled participants and of the patient group (Table 2) were best fitted by Models 1 and 3. These two models included two sensitivity parameters for rewards and punishments, which were used to scale the prediction error, a lapse and a decay parameter capturing forgetting in choice behaviour. In absolute terms, Model 1 fitted the data best, but it was not significantly different from Model 3. The only difference between Models 1 and 3 designs lied in their learning rates: Model 1 had two learning rates (*α* and *β*) that were added for updating reward values (reward LR=α+β) and subtracted for updating punishment values (punishment LR=α−β), whereas Model 3 had separate, fixed learning rates for rewards and punishments. In the control group, Model 3 showed the best absolute fit, but it was not significantly different from the other models we tested.

**Table 2:** Model comparison for the four-armed bandit task

Group	Model	ELPD difference	SE difference	Sigma effect	LOOIC
All	1	0	0	–	24386.3539
	3	−53.422223	37.8139003	−1.4127668	24493.1983
	2	−276.00621	90.2032862	−3.0598243	24938.3663
	4	−280.33323	91.9252409	−3.0495785	24947.0203
	5	−336.24668	97.0027097	−3.4663638	25058.8472
Arthritis	1	0	0	–	12754.9737
	3	−46.954621	37.2017901	−1.2621603	12848.8829
	4	−221.65393	80.4995507	−2.7534803	13198.2815
	2	−226.78714	77.0709632	−2.9425757	13208.5479
	5	−264.26755	83.3235728	−3.171582	13283.5088
Controls	3	0	0	–	11667.014
	1	−2.6956325	9.89283891	−0.2724832	11672.4052
	4	−46.800276	48.2061096	−0.970837	11760.6145
	2	−48.534824	49.9225293	−0.9722028	11764.0836
	5	−56.156688	49.6040047	−1.1320999	11779.3273

Five models were fitted to all participants, and to the arthritis and control groups separately: (1) differential learning rates (LR), lapse and decay parameters; (2) differential LR with lapse parameter, no decay; (3) separate LRs for rewards and punishments, lapse and decay parameters; (4) separate LRs for rewards and punishments, lapses but no decay; and (5) separate LRs for rewards and punishments, without lapse or decay parameters. We report the expected log predictive density (ELPD) difference between the best-performing model [lowest leave-one-out cross-validation information criterion (LOOIC)] and each model, alongside the standard error (SE) of the difference and the sigma effect (the ratio between the ELPD and SE difference, which is a proxy for significance).

Our main interest was to evaluate group differences of model parameters. Hence, we compared the group estimates of the parameters of Model 1 between the arthritis patient group and the control group, to investigate any core underlying differences in choice behaviour.

**Figure 2:**
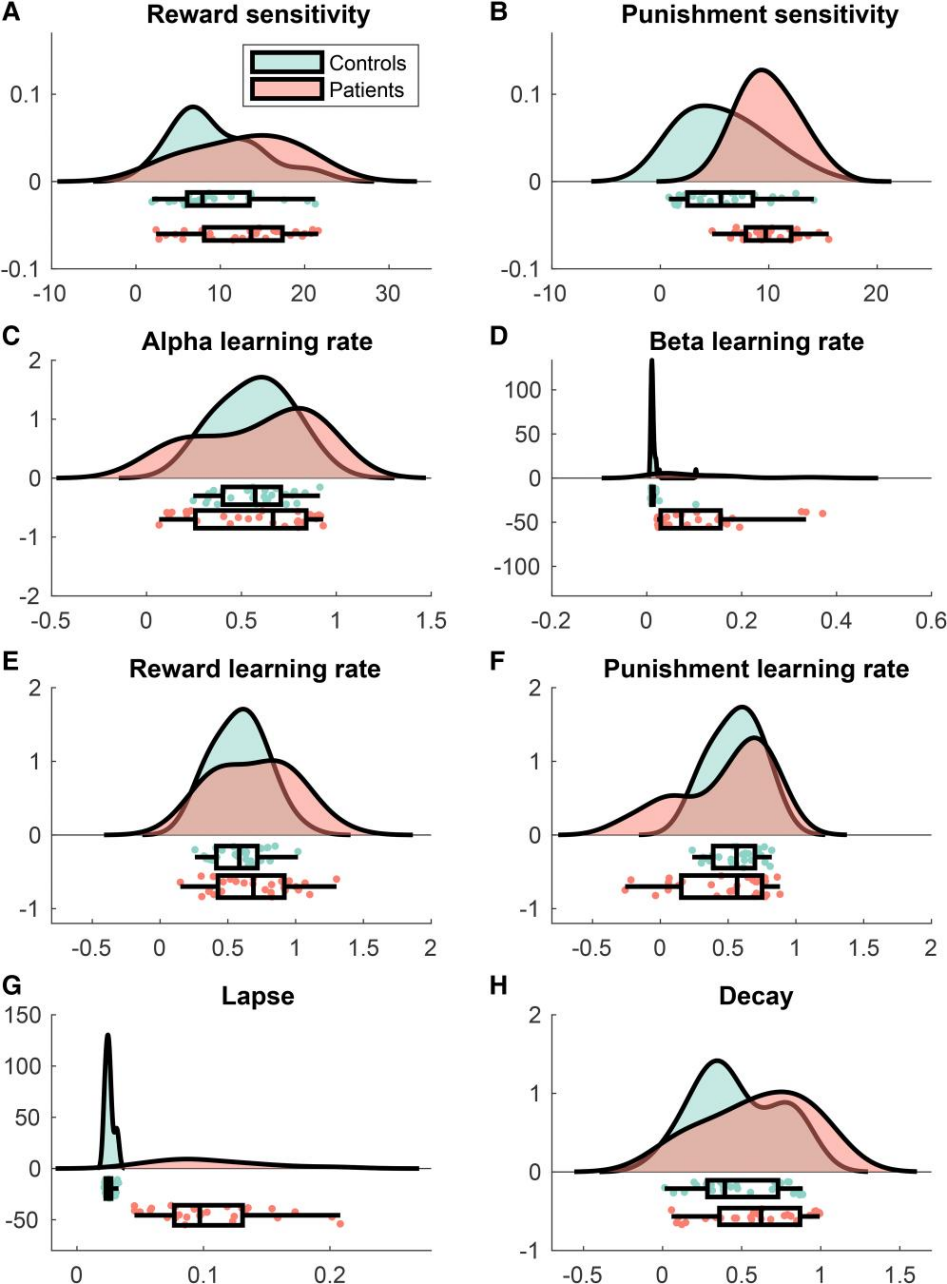
**Participant’s estimates of each parameter**: (A) reward sensitivity; (B) punishment sensitivity; (C) Alpha learning rate; (D) beta learning rate; (E) reward learning rate; (F) punishment learning rate; (G) lapse; and (H) decay. For each parameter (in arbitrary units), we show the raincloud plots, which include the (half) probability distribution function, scatter plots and box plots of the data of arthritis patients (red) and controls (green).

The group-level estimates of each free parameter of Model 1 are reported in Table 3, and the estimates for individual participants are shown in Fig. 2. In keeping with our core hypothesis, participants with chronic arthritis were more sensitive to punishments when compared with controls, i.e. showed enhanced punishment sensitivity (P). In addition, we also found that they had faster memory decay and greater lapses in the choice behaviour. Finally, the differential component of the learning rate (*β*) was also higher in patients.

**Table 3:** Comparisons of Model 1 parameters between arthritis and control groups

Parameter	Arthritis	Controls	Lower 95% HDI	Upper 95% HDI
Reward sensitivity	12.750 (5.911)	9.417 (5.031)	−1.004	7.695
Punishment sensitivity	9.953 (2.577)	5.778 (3.715)	1.388*	8.016*
*α*	0.585 (0.300)	0.569 (0.178)	−0.133	0.183
*β*	0.106 (0.100)	0.016 (0.018)	0.0130*	0.101*
Reward learning rate (*α* + *β*)	0.679 (0.069)	0.590 (0.043)	−0.067	0.25
Punishment learning rate (*α* − *β*)	0.547 (0.074)	0.570 (0.047)	−0.199	0.142
Lapse *ξ*	0.105 (0.043)	0.026 (0.003)	0.036*	0.105*
Decay	0.603 (0.311)	0.469 (0.261)	0.008*	0.403*

Group-level parameter estimates for the arthritis and control groups (mean and standard deviation) in Model 3 (separately fitted for each group) and high-density interval (HDI) group differences (lower and upper 95% HDI).

*Significant differences do not include zero.

### Neural correlates of punishment prediction error

**Figure 3:**
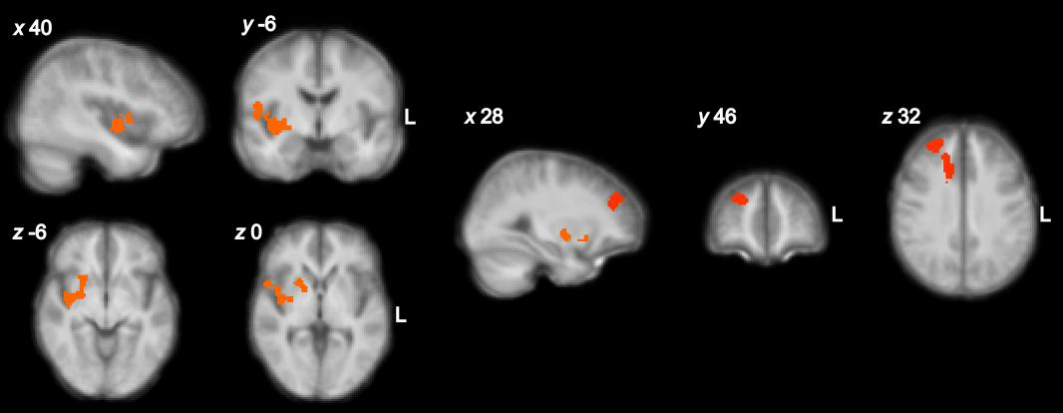
**Increased neural processing of the punishment prediction error in the arthritis group versus controls, modulated by punishment sensitivity.** Increased blood oxygenation level-dependent responses associated with PPE in the right posterior insula, putamen, pallidum and the dorsolateral prefrontal cortex. The colour scale represents *z*-scores ranging from 0 to 5, thresholded at *z* > 2.6, *P* < 0.05. The statistical contrast was overlaid over a structural brain image, obtained by averaging the high-resolution structural images of the study participants.

Participants performed the bandit task whilst undergoing functional brain MRI. We aimed to identify brain changes associated with reward and loss decision-making. Given that punishment sensitivity scales the magnitude of the value of the punishment as used for learning avoidance actions, this is reflected in brain responses correlated with the punishment prediction error (PPE), with the individually estimated punishment sensitivity as a covariate (the punishment sensitivity is an estimated constant that scales the prediction error in our reinforcement learning models). Thus, we evaluated whether the neural activity associated with the PPE, and modulated by punishment sensitivity, differed across groups. The arthritis group showed increased activity in the right posterior insular cortex, putamen, pallidum and dorsolateral prefrontal cortex (Fig. 3 and Supplementary Table 6). There was no significant cluster of decreased activity at the cluster threshold used (*z* > 2.6, *P* < 0.05). For completeness, common activity across the two groups is reported in the Supplementary material, ‘Imaging Results’, Supplementary Fig. 3 and Supplementary Table 8.

### Neural correlates of reward prediction error

**Figure 4:**
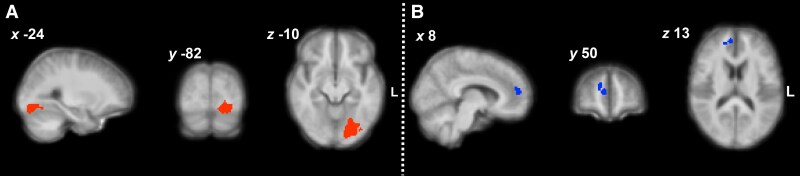
**Increased (red; A) and decreased (blue; B) activity associated with the reward prediction error in the arthritis group versus controls.** Colour scale represents *z*-scores ranging from 0 to 5, thresholded at *z* > 2.6, *P* < 0.05. The statistical contrast was overlaid over a structural brain image, obtained by averaging the high-resolution structural images of the study participants.

Reward prediction error (RPE) activity modulated by reward sensitivity (a constant) was decreased in arthritis participants versus controls in a rostral region of the paracingulate gyrus and neighbouring white matter and increased in the left occipital fusiform gyrus, area V4 (Fig. 4 and Supplementary Table 7). There was no significant cluster of increased activity at the cluster threshold used (*z* > 2.6, *P* < 0.05). Common activity across groups is reported in Supplementary Fig. 4 and Supplementary Table 9.

### Connectivity analyses

**Figure 5:**
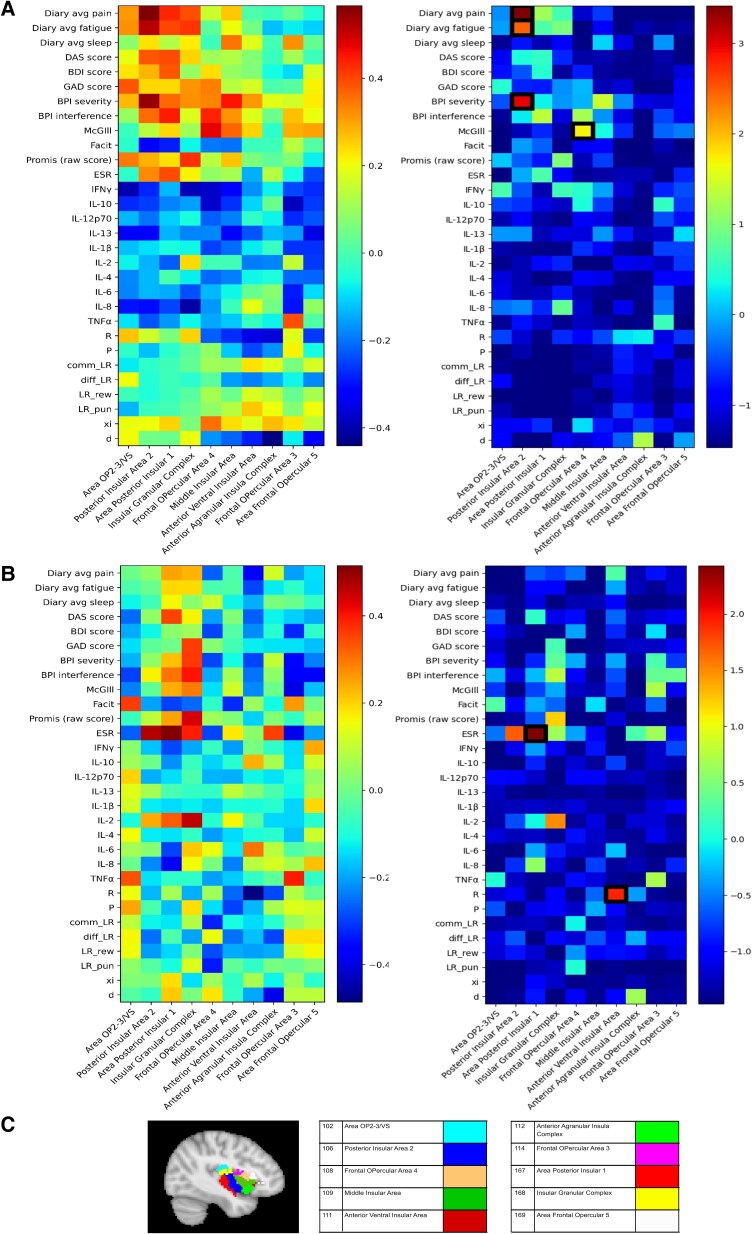
**Connectivity results.** (**A**) Bayesian Pearson correlation of degree centrality of 10 insular nodes with clinical outcomes and model parameters for the patient group in task functional MRI (fMRI). Black lines in the log Bayes factors (logBF10) plots highlight significant correlations that satisfy the false discovery rate (FDR) threshold of 0.01. (**B**) Bayesian Pearson correlation of degree centrality of 10 insular nodes with clinical outcomes and model parameters for the patient group in resting-state fMRI. Significant correlations after FDR corrections: area post insular 1 with ESR (Pearson’s *r* = 0.515, logBF10 = 2.43) and anterior ventral insular area with reward sensitivity (Pearson’s *r* = −0.486, logBF10 = 1.935). (**C**) A colour-coded map of insular nodes derived from the Glasser atlas (number denotes the parcel number in the Glasser atlas).

The correlation of punishment sensitivity with evoked blood oxygenation level-dependent (BOLD) responses in the insula is in keeping with the core hypothesis that the insula is a central part of a behavioural homeostatic network that mediates motivational changes in the pain state. To look at this further, we aimed to identify whether the insula connectivity during the task is correlated with pain behaviour and task parameters. To do this, we calculated the network centrality (here, degree centrality), which captures the importance or influence of the insula as a network hub during the task. We parcellated the brain into 180 symmetrical regions of interest using a functional (Glasser) brain atlas and constructed a binarized connectivity network using thresholded pairwise Pearson correlations (see the ‘Materials and methods’ section). This analysis ignores the task events and simply considers overall connectivity during the task as a whole. We found that degree centrality in posterior insula subregions was correlated with subjective reports of daily average pain and fatigue scores (Fig. 5A). Following Bayesian FDR correction with a threshold of 0.01, there was evidence for a correlation between posterior insular area 2 and measures of pain (average daily pain rating from weekly diaries: Pearson’s *r* = 0.566, LogBF10 = 3.413; Brief Pain Inventory (BPI) severity score: Pearson’s *r* = 0.544, LogBF10 = 2.958) and fatigue (average daily fatigue rating from weekly diaries: Pearson’s *r* = 0.517, LogBF10 = 2.468) and between frontal opercular area 4 and McGill questionnaire (Pearson’s *r* = 0.472, LogBF10 = 1.711). These significant correlations are highlighted with black borders in LogBF10 plots. These correlations were less apparent at rest, i.e. when performing the same analysis using the resting state fMRI data rather than task fMRI data (Fig. 5B), in line with previous work.^31^ The analysis of connectivity during instrumental learning provides evidence that posterior insula activity is not only correlated with pain-related changes in objective task behaviour, but also that it sits within a network of regions whose activity is correlated with subjective ratings of fatigue and ongoing pain.

Finally, we also explored a range of graph theory measures (Supplementary Figs 5–8). Explorative analyses show that closeness and eigenvector centralities are correlated more with outcome measures in the task fMRI setting, in comparison to betweenness and load centrality measures. Degree centrality was selected for the main analysis because it provided the most interpretable results in relationship to the study hypotheses. Other measures yielded similar but less informative patterns.

## Discussion

The results of this exploratory study reveal a computational signature of chronic pain and fatigue, reflected in the pattern of changes in the underlying parameters that govern motivated behaviour. At the heart of this signature is an increase in punishment sensitivity, which demonstrates a generalized form of hyper-protective behaviour that enhances learning of aversive outcomes and might lead to excessive avoidance. This might allow the organism to enhance defence beyond transient pain, which can be mediated by central and peripheral hypersensitivity, to other aversive outcomes.

In our reinforcement learning model, punishment sensitivity was a constant that scaled the punishment prediction errors (PPE) at each time point, whilst the PPE reflected the change of punishment value at each trial, which is used for learning. Punishment prediction errors, covaried by the punishment sensitivity constants, were used as trial-by-trial regressors of neural activity. We found increased PPE-related activity in the right posterior insular cortex, putamen, pallidum and dorsolateral prefrontal cortex (Fig. 3), in the inflammatory arthritis group. Finally, we computed the centrality^32^ of insula networks to capture the importance of different insula subregions as a network hub during instrumental learning. We found that posterior insula area 2 activity was correlated with subjective reports of daily fatigue and pain scores, highlighting its clinical significance. Importantly, these findings remain robust even after applying a conservative Bayesian FDR correction to control for multiple comparisons, reducing the likelihood of false-positive correlations.

In previous instrumental learning studies on healthy participants, PPE activity was reported in the anterior insula,^33^ putamen^34^ and dorsolateral prefrontal cortex.^33^ The role of the insula is in keeping with the hypothesis that it plays a fundamental role in pain^16,35-37^ and fatigue states.^38,39^ This can also be related to a proposed role in active inference of interoceptive,^40,41^ somatosensory^42^ and nociceptive signals.^15,16,18^ The insula is well placed to interface afferent nociceptive information with efferent control through connectivity to mesolimbic and mesocortical pathways, other cortical sites involved in learning and motivation (such as the anterior cingulate and medial prefrontal cortex) and descending pain control systems.^9,19^

We also note that the importance of the insular networks was much more apparent during task performance than at rest. Although this finding needs to be cross-validated in new datasets, it might suggest that looking at brain connectivity in the context of motivated behaviour might be more sensitive than pure resting state brain activity, when used for biomarker generation. We should also note that we restricted our analysis to the insula, based on our *a priori* hypothesis, but it might well be that other regions (such as prefrontal cortex and anterior cingulate cortex) show symptom-correlated network activity.

The computational signature of instrumental learning in inflammatory arthritis also includes other components of behaviour, including the learning rate differential between rewards and punishments, alongside increased forgetting and choice lapses. This specific pattern appears to be distinct from that seen in other clinical conditions, using the same or similar task. For instance, fibromyalgia is associated with decreased brain responses to rewards and punishments^43^ and a more persistent punishment memory.^44^ Depression is also associated with a reduced sensitivity to rewards^11^. In contrast, increased punishment learning rates are reported in anxiety,^10^ opposite to the relative increase in reward learning rate seen here, suggesting more dynamic updating of rewards in comparison to punishments. The increased sensitivity to punishments in inflammatory arthritis is also different from the reduced general outcome sensitivity observed in Parkinson’s disease as the result of deep brain stimulation^14^ and the reduced reward outcome value reported following tryptophan depletion.^45^ An increase in the lapse and decay rates was also found in Parkinson’s patients, as in our study. This adds randomness to the decisions and probably reflects a non-specific, disruptive effect of pain and fatigue on attention. Together, this illustrates how the chronic pain and fatigue state seen in inflammatory arthritis is computationally distinct from depression, anxiety and Parkinson’s disease.

The behavioural manifestation of computational components of learning and decision-making maps well to the predictions of chronic pain models. Notably, increased punishment sensitivity is a central prediction of the fear avoidance model.^2,3^ Previous investigations have focused on punishment generalization,^46^ a distinct component of avoidance in which aversive values spread to perceptually similar pain predictors. Importantly, both increased punishment sensitivity and aversive generalization drive the same tendency towards enhanced avoidance. This is a critical component of mechanistic models of chronic pain, which posit that the brain forms a hierarchical internal representation of the injury to drive protective and recuperative behaviours.^9^ Although greater avoidance reduces the chance of worsening damage through new actions, it inherently affects the opportunity to learn whether an injury has resolved (information restriction) and will tend to perpetuate internal representations of injury states.^5^ Future longitudinal studies would need to determine whether punishment sensitivity is a manifestation of the pain state or an outcome predictor.

## Conclusion

Clinically, pain and fatigue are often highly correlated in inflammatory chronic pain conditions.^47^ However, the methods used in this study could be applied to painless, fatigue-dominant disorders, such as chronic fatigue syndrome and long COVID, to dissect the effect of fatigue from that of pain on instrumental learning. In inflammatory arthritis, the two are nearly impossible to disentangle. The approach here provides a behaviourally and computationally informed approach to disentangling these in future studies across other pain and fatigue cohorts.

## Supplementary Material

awae408_Supplementary_Data

## Data Availability

All code, raw behavioural, clinical and demographical data and group statistical maps are available open source: https://osf.io/82hp9/. Raw MRI images are available upon reasonable request to the authors.
