# Supplementary material for: Enhanced behavioural and neural sensitivity to punishments in chronic pain and fatigue
Source: Brain. 2024 Dec 30;148(6):2151–62. doi: 10.1093/brain/awae408 (PMC12129725; doi:10.1093/brain/awae408)
Supplement: awae408_Supplementary_Data [file awae408_supplementary_data.zip › FAPIA_for_Brain__revision__supplementary.pdf]

Supplementary Materials

Clinical and demographic data

Clinical and demographic data of the 57 participants who completed the full study are reported in Table S1. As expected, patients scored higher than controls on the Brief Pain Inventory (severity score: t55=11.188, p<0.001, Cohen's d=1.860, BF10=4.495x10$^{(}$ + 12); interference score: t55=8.074, p<0.001, BF10=9.935x10$^{(}$ + 7)), McGill Pain Questionnaire (t55=7.807, p<0.001, Cohen's d=2.068, BF10=3.873x10$^{(}$ + 7)). Patients reported more fatigue (FACIT scale: t55=-9.055, p<0.001, Cohen's d=-2.399, BF10=3.132e+9) and poorer sleep quality than controls (PROMIS sleep scale: t55=4.784, p<0.001, Cohen's d=1.268, BF10=1302.373). This is paralleled by higher mean ratings of pain (t55=12.299, p<0.001, Cohen's d=3.259, BF10=1.650e+14) and fatigue (t55=8.672, p<0.001, Cohen's d=2.298, BF10=8.177e+8), and lower ratings of sleep quality (t55=2.948, p=0.005, Cohen's d=0.788, BF10=8.667), collected using diaries over a continuous period of 5 days, relative to controls. Average levels of pain and fatigue were highly correlated across participants (Pearson's r=0.84, BF10=2.210x10^{1}3). Interestingly, patients adopted higher severity ratings to describe their fatigue levels relative to their pain levels (mean fatigue diary rating ± SD, 5.461 ± 1.765; mean pain diary rating ± SD, 4.804 ± 1.698; t28=2.780, p=0.010, Cohen's d=0.516, BF10=4.710). Unsurprisingly, patients consumed more caffeine per day (t55=3.444, p=0.001, Cohen's d=0.913, BF10=28.578), but comparable units of alcohol than controls (t55=0.221, p=0.826, Cohen's d=0.059, BF10=0.274). Finally, patients reported greater depression symptoms at the Beck Depression Inventory (mean score ± SD, 8.429±5.295; group difference, t55=6.961, p<0.001, Cohen's d=1.860, BF10=1.798e+6) and generalised anxiety symptoms at the GAD7 test (mean score ± SD, 7.857±4.897; group difference, t55=6.560, p<0.001, Cohen's d=1.753, BF10=448796.707). Overall, we found strong evidence for associations between pain and fatigue questionnaires (BPI, McGIll and FACIT) and self-reported measures of depression and generalised anxiety (all correlations $p < 0.001$).

Table S1: Composition of the sample

ID	Group	Sex	Age	Diagnosis	BPI severity	McGIll	Pain diary	Facit	Fatigue diary	BDI	GAD	Medication
P01	Patient	F	52	RA	6.50	23	7.307692	27.000000	7.307692	2.0	4.0	–
P02	Patient	F	56	RA	5.25	13	5.076923	36.000000	6.272727	7.0	16.0	Adalimumab (inj/2 weeks), Hydroxychloroquine...
P03	Patient	F	50	RA	3.25	9	4.000000	38.000000	3.727273	6.0	8.0	Methotrexate (15mg/week)
P05	Patient	M	60	RA	2.25	4	1.985915	29.545455	4.549296	4.0	5.0	NaN
P07	Patient	F	36	RA	7.00	20	7.100000	12.000000	7.000000	19.0	15.0	Etanercept (50mg inj/week), Leflunomide (20mg/d...
P08	Control	M	60	NaN	0.25	0	0.428571	49.000000	2.045455	0.0	0.0	NaN
P10	Control	F	66	NaN	0.00	0	0.454545	52.000000	1.909091	0.0	0.0	NaN
P12	Control	F	43	NaN	2.75	8	1.333333	28.000000	3.933333	3.0	1.0	NaN
P13	Control	M	55	NaN	0.25	0	0.642857	46.000000	0.642857	1.0	0.0	NaN
P14	Control	F	65	NaN	0.00	0	0.000000	43.000000	0.666667	1.0	4.0	NaN
P15	Control	F	62	NaN	0.00	0	0.000000	47.000000	3.538462	0.0	4.0	NaN
P16	Patient	F	63	RA	1.25	2	2.000000	33.000000	2.933333	NaN	NaN	Hydroxychloroquine (200mg/12h)
P17	Patient	F	55	RA	1.00	2	2.333333	49.000000	2.933333	2.0	6.0	Methotrexate (10mg/week), Sulfasalazine (500mg/...
P18	Patient	F	37	RA	4.75	22	4.866667	28.000000	2.066667	5.0	9.0	Methotrexate (15mg/week), Adalimumab (5mg inj/2...
P19	Patient	F	59	RA	3.00	13	3.000000	40.000000	2.533333	1.0	2.0	NaN
P20	Control	F	50	NaN	0.00	0	2.255721	47.000000	2.357143	0.0	1.0	NaN
P23	Control	M	51	NaN	0.75	4	0.714286	43.000000	1.142857	0.0	2.0	NaN
P24	Patient	F	54	RA	2.50	6	3.533333	41.000000	3.333333	10.0	5.0	Hydroxychloroquine (200mg/12h), Methotrexate (2...
P27	Patient	F	56	RA	1.50	4	2.400000	23.000000	4.333333	5.0	2.0	Adalimumab (inj/2 weeks), Methotrexate (15mg/we...
P28	Patient	F	65	RA	4.75	11	4.733333	29.250000	6.800000	12.0	18.0	Methotrexate (15mg/week), Hydroxychloroquine (2...
P29	Patient	F	37	RA	2.50	12	3.421053	33.000000	4.684211	5.0	6.0	Anti TNF B (inj/week), Methotrexate (7.5mg/week...
P32	Control	M	51	NaN	0.00	0	0.000000	52.000000	2.071429	0.0	0.0	NaN
P33	Patient	F	47	RA	3.50	3	4.600000	36.000000	5.800000	6.0	6.0	Adalimumab (inj/2 weeks), Leflunomide (20mg/day)
P34	Patient	F	57	RA	6.00	17	7.800000	7.000000	7.916667	6.0	15.0	Golimumab (inj/month), Methotrexate (15mg/week)
P35	Patient	F	54	RA	4.25	21	4.466667	16.000000	4.066667	22.0	14.0	Methotrexate (25mg/week), Naproxen (500mg/12h),...
P37	Control	F	50	NaN	0.00	0	0.000000	52.000000	1.230769	0.0	0.0	NaN
P38	Patient	F	60	RA	5.00	17	6.090909	22.000000	5.666667	9.0	5.0	Benepali (50mg/weekly), Leflunomide (20mg/day),...
P40	Patient	F	57	RA	5.75	4	5.692308	34.000000	7.000000	5.0	9.0	Tofacitinib (5mg/12h)
P42	Patient	F	53	PsA	3.75	10	3.846154	33.000000	4.230769	14.0	6.0	Amitriptyline (75mg/day), Amlodipine (10mg/day)...
P43	Control	F	33	NaN	1.00	0	1.200000	49.000000	1.733333	5.0	2.0	NaN
P44	Patient	F	35	RA	6.00	17	3.357143	31.000000	4.769231	11.0	5.0	NaN
P46	Control	M	42	NaN	0.00	0	0.000000	50.000000	2.133333	1.0	0.0	NaN
P47	Control	F	67	NaN	1.00	4	0.642857	46.000000	1.642857	4.0	3.0	NaN
P49	Control	F	38	NaN	0.00	0	0.166667	52.000000	1.916667	0.0	0.0	NaN
P50	Control	M	69	NaN	0.00	0	0.000000	45.000000	3.214286	0.0	0.0	NaN
P54	Patient	F	52	RA	6.75	20	4.769231	21.000000	7.769231	10.0	16.0	Methotrexate (25mg/week), Sulfasalazine (500mg/...
P55	Control	F	68	NaN	1.00	2	1.533333	48.000000	2.800000	5.0	5.0	NaN
P56	Patient	F	45	RA	6.75	26	6.466667	14.000000	5.733333	13.0	13.0	Prednisalone (2.5mg/day), Nortriptyline (10mg/day)
P57	Patient	F	54	RA	3.25	15	4.937500	27.000000	6.375000	4.0	0.0	Hydroxychloroquine (200mg/day), Methotrexate (1...
P58	Control	F	59	NaN	0.00	0	0.600000	52.000000	0.733333	4.0	6.0	NaN
P59	Control	M	65	NaN	0.00	0	0.181818	50.000000	1.454545	0.0	0.0	NaN
P60	Control	F	64	NaN	0.00	0	0.071429	49.000000	0.285714	1.0	1.0	NaN

Continued on next page

ID	Group	Sex	Age	Diagnosis	BPI severity	McGIll	Pain diary	Facit	Fatigue diary	BDI	GAD	Medication
P61	Patient	M	45	PsA	5.25	21	5.600000	32.000000	8.300000	8.0	10.0	Meloxicam (25mg/day), Methotrexate (25mg/week)
P64	Patient	F	49	RA	4.25	4	5.461538	32.000000	6.307692	4.0	3.0	Methotrexate (25mg/week), Benepali, Etanercept ...
P65	Patient	F	62	PsA	6.25	11	7.642857	25.000000	6.214286	6.0	5.0	Methotrexate (10mg/week)
P66	Patient	F	62	RA	3.75	24	5.000000	3.000000	6.600000	16.0	5.0	Methotrexate (15mg/week)
P68	Control	F	58	NaN	0.00	0	0.000000	42.000000	1.250000	0.0	0.0	NaN
P69	Patient	F	39	RA	7.00	19	7.500000	11.000000	8.000000	16.0	6.0	Methotrexate (10mg/week), Tocilizumab (inj/week)
P71	Control	F	42	NaN	0.00	0	0.000000	50.000000	0.800000	2.0	2.0	NaN
P72	Control	M	54	NaN	0.00	0	0.000000	48.000000	4.285714	0.0	0.0	NaN
P73	Patient	F	45	PsA	3.00	2	4.333333	34.000000	5.133333	8.0	6.0	Methotrexate (20mg/week)
P74	Control	M	44	NaN	0.00	0	0.000000	51.000000	1.571429	0.0	0.0	NaN
P75	Control	F	61	NaN	1.50	9	2.166667	48.000000	0.833333	2.0	0.0	NaN
P76	Control	F	46	NaN	0.00	0	0.000000	45.000000	5.466667	2.0	2.0	NaN
P78	Control	M	39	NaN	0.00	0	2.100000	50.000000	2.700000	1.0	2.0	NaN
P79	Control	F	48	NaN	0.00	0	0.000000	47.000000	1.000000	0.0	4.0	NaN
P80	Control	F	57	NaN	0.00	0	0.000000	52.000000	0.000000	0.0	0.0	NaN

Additional task results

Patients won a comparable amount of money (net £4.62±2.50) than controls (net £5.18±2.53) in the 4-armed bandit task (t55=0.837, p=0.406, Cohen's d=0.222, BF10=0.359). Reaction times were comparable between groups (t55=0.794, p=0.430, Cohen's d=0.210, BF10=0.349).

Patients and controls were similarly likely to switch choice, irrespective of the outcome (t55=0.528, p=0.6, Cohen's d=0.140, BF10=0.301). However, patients were overall slower at making a choice than controls (p=0.013) and we found a nominally-significant interaction between group and choice type (2 levels: repeated, switched; p=0.027); the choice to switch was slower in patients than controls (t55=2.478, p=0.016, Cohen's d=0.657, BF10=3.243), whereas the choice to stick to the previous decision had similar latency in the two groups (t55=1.484, p=0.144, Cohen's d=0.393, BF10=0.667).

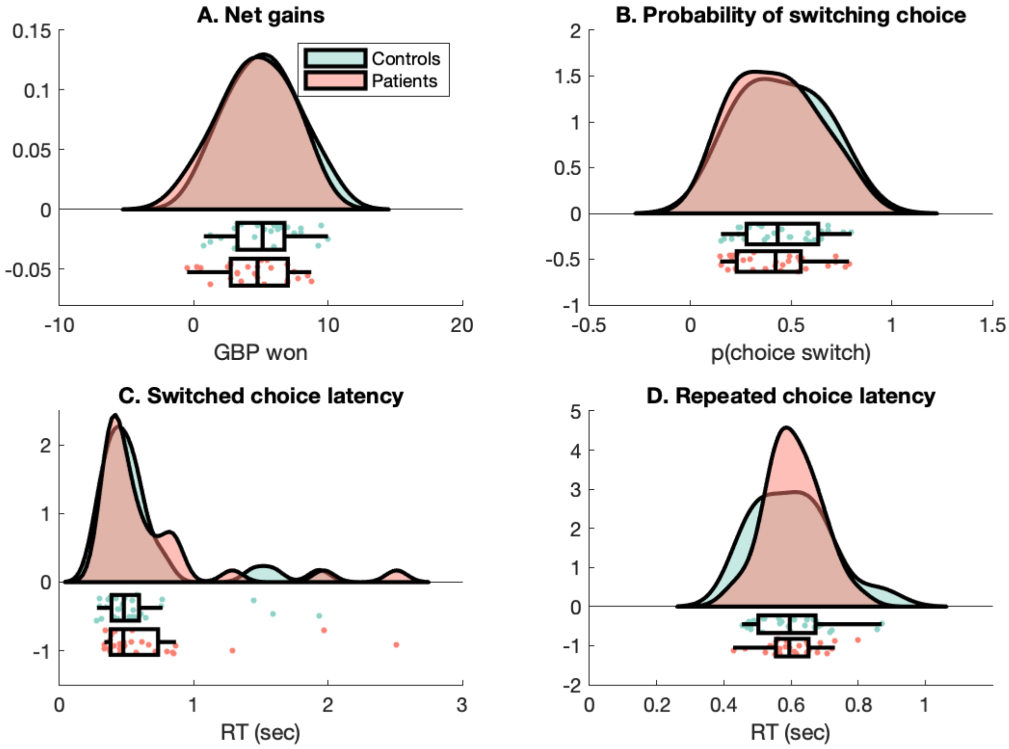

Figure S1: **Task performance.** A. Net gains (GBP); B. Probability of switching choice irrespective of the outcome; C. Latency of the switched choice; D. Latency of the repeated choice in controls (green) and patients (red). For each variable, the probability density functions, scatterplot of individual data and boxplot are shown.

We evaluated whether the frequency of choice switches was affected by the outcome of the preceding trial (4 levels: reward only, punishment only, simultaneous reward and punishment, no outcome) and by the diagnosis (2 levels: inflammatory arthritis, healthy controls). A mixed-effects ANOVA provided evidence for an effect of condition (F1.45,79.75=110.824, p<0.001, eta2=0.637), but no evidence for main effect of group (F1,55=0.907, p=0.345, eta2=0.016) nor interaction (F1.45,79.75=0.517, p=0.540, eta2=0.003). Irrespective of their group, participants were more likely to change their choice following a trial that yielded either a loss or no outcome than in trials that yielded either a win or a simultaneous win and loss (all ps < 0.001, Cohen's d > 1.5). Moreover, the frequency of choice switches was similar after a loss and no outcome (p=0.062, Cohen's d=0.253); participants switched choices less frequently after affording a win rather than an ambiguous outcome

(p=0.017, Cohen's d=0.360). Finally, the total frequency of choice switches were comparable across groups (t55=0.952, p=0.345, Cohen's d=0.252, BF10=0.391).

Sleep Actigraphy

We used ActiLife v6.13.3 to extract sleep measures. Automatic detection of sleep periods was implemented with Tudor-Locke automatic sleep period detection. If there was a discrepancy of >2 hours between the automatic detected timing and the self-documented bedtime or out-of-bed time, the self-documented times would be entered manually as sleep periods. The Cole-Kripke algorithm was applied to the data to determine sleep parameters cole1992automatic. Data from 5 participants (3 patients) with <5 nights of recording were excluded from further analyses, leaving 52 participants for analysis (25 patients).

Controls and patients showed comparable results in the sleep parameters, total sleep time (t(50)=1.597, p=.117, Cohen's d=0.443, BF10=0.788), movement index (t(50=-1.684, p=0.098, Cohen's d=0.467, BF10=0.884), sleep fragmentation (t(50)=-1.648, p=0.106, Cohen's d=0.457, BF10=0.842), sleep latency (t(50)=-0.296, p=0.768, Cohen's d=0.082, BF10=0.289), sleep efficiency (t(50)=0.794, p=0.431, Cohen's d=0.220, BF10=0.361) and wake-after-sleep-onset (t(50)=-0.171, p = 0.865, Cohen's d=0.047, BF10=0.282).

Daily activity

We used ActiLife v6.13.3 to extract daily activity measures, implementing the default wear time validation and freedson1998calibration algorithm to detect activity bouts. Days with < 1080 epochs (i.e., 18 hours) of data were excluded from the analyses (154 days removed in total). Similar to the sleep analysis, any participants with < 5 days of recording were removed from further analysis (N=5, 2 patients), leaving 52 participants for analysis (26 patients).

Patients and controls showed comparable results in the activity parameters, with mean daily steps (mean ±SD, patients: 12114.340 ±4323.304, controls: 14191.425 ± 3714.191; t(50)= 1.858, p=.069, Cohen's d=0.515, BF10=1.131), proportion spent in sedentary activity (mean ± SD, patients: 0.536 ± 0.108, controls: 0.498 ±0.098; t(50)=-1.347, p=.184, Cohen's d=0.374, BF10=0.585), proportion spent in light activity (mean ± SD, patients: 0.355 ± 0.083, controls: 0.371 ± 0.072; t(50)=0.768, p=.446, Cohen's d=0.213, BF10=0.355), proportion spent in moderate activity (mean ± SD, patients: 0.109 ± 0.062, controls: 0.131 ± 0.043; t(50)=1.487, p=.143, Cohen's d=0.413, BF10=0.688), neither group spending any time in vigorous activity, number of bouts (mean ± SD, patients: 8.371 ± 5.703, controls: 9.667 ± 4.585; t(50)=0.903, p=.371, Cohen's d=0.251, BF10=0.389), total time of bouts (mean ± SD, patients: 137.213 ± 100.688, controls: 162.494 ±77.400; t(50)=1.015, p=.315, Cohen's d=0.282, BF10=0.425), and total activity bout count (mean ± SD, patients: 462028.583 ± 350630.645, controls: 596035.778 ± 253689.269; t(50=1.579, p=.121, Cohen's d=0.438, BF10=0.770).

Supplementary modelling analyses

0.1 Parameter recovery

To asses the reliability of our modelling analysis, for each model we performed parameter recovery analysis, where we simulated participants' responses using newly drawn individual-level parameters from the group-level distributions, for each group and for a pooled dataset.

We then fit the same model to the simulated data and calculated Pearson correlation coefficients r between the generated and estimated individual-level parameters. We recovered each individual parameter (for each member of the population dataset) 50 times and calculated the mean and SD of the correlation between the true and recovered parameter values. See results in S2-S4. The higher the coefficient r, the more reliable the estimates are, which can be categorised as: poor (if r <0.5); fair (if 0.5< r <0.75); good (0.75< r <0.9); excellent (if r >0.9) white$_t esting_2$018.

Table S2: Pearson correlation coefficients (SD) for parameter recovery analysis for each parameter from five RL models for a pooled dataset.

Model ID	LR1	LR2	R	P	ξ	d
1	0.91 (0.03)	0.75 (0.09)	0.87 (0.04)	0.78 (0.06)	0.67 (0.08)	0.92 (0.02)
2	0.90 (0.11)	0.75 (0.16)	0.83 (0.09)	0.79 (0.13)	0.61 (0.17)	
3	0.92 (0.02)	0.26 (0.11)	0.85 (0.04)	0.91 (0.03)	0.65 (0.10)	0.92 (0.02)
4	0.95 (0.02)	0.88 (0.04)	0.88 (0.03)	0.90 (0.03)	0.70 (0.11)	
5	0.95 (0.02)	0.88 (0.03)	0.94 (0.02)	0.92 (0.03)		

Table S3: Pearson correlation coefficients (SD) for parameter recovery analysis for each parameter from five RL models for patients.

Model ID	LR1	LR2	R	P	ξ	d
1	0.93 (0.03)	0.78 (0.11)	0.84 (0.05)	0.61 (0.11)	0.76 (0.09)	0.93 (0.03)
2	0.90 (0.11)	0.73 (0.20)	0.76 (0.16)	0.73 (0.17)	0.70 (0.19)	
3	0.90 (0.06)	0.20 (0.16)	0.83 (0.06)	0.90 (0.04)	0.69 (0.10)	0.91 (0.05)
4	0.96 (0.02)	0.91 (0.05)	0.80 (0.07)	0.81 (0.07)	0.73 (0.11)	
5	0.95 (0.03)	0.91 (0.04)	0.94 (0.03)	0.86 (0.05)		

Table S4: Pearson correlation coefficients (SD) for parameter recovery analysis for each parameter from five RL models for healthy controls.

Model ID	LR1	LR2	R	P	ξ	d
1	0.90 (0.05)	0.14 (0.22)	0.92 (0.04)	0.92 (0.03)	0.29 (0.16)	0.90 (0.04)
2	0.88 (0.11)	0.72 (0.17)	0.88 (0.11)	0.88 (0.08)	0.27 (0.25)	
3	0.91 (0.04)	0.22 (0.18)	0.91 (0.03)	0.93 (0.03)	0.23 (0.18)	0.89 (0.05)
4	0.92 (0.03)	0.83 (0.07)	0.94 (0.04)	0.94 (0.02)	0.26 (0.17)	
5	0.92 (0.03)	0.80 (0.10)	0.95 (0.02)	0.94 (0.03)		

Moreover, to assess the number of simulations needed, we calculated the average error of the parameter recovery correlation (and its error) for each model, parameter, and dataset as a function of increasing number of simulation, as plotted in Figure S2. The average was obtained from the 1000 randomly chosen permutations of different simulations at each n (out of 50).

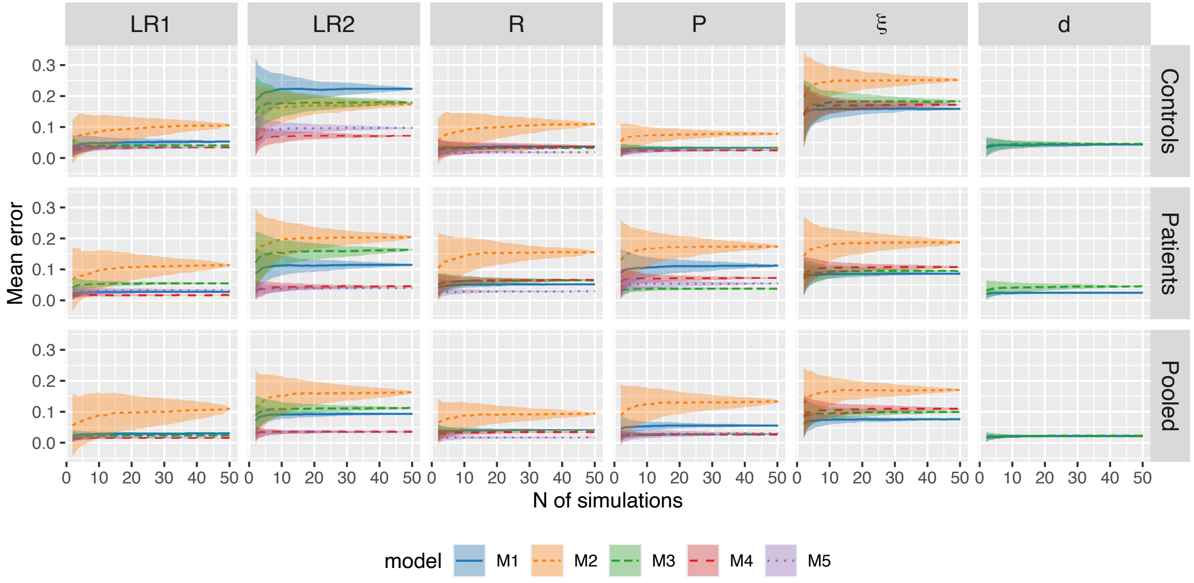

Figure S2: **Parameter recovery mean error** of the correlation for each model and each parameter. The average error is plotted as a function of simulation number averaged across 1000 permutations of 50 simulations.

0.2 Model recovery

We also performed model recovery analysis, where we first simulated responses using each model and then fit each model to the model-specific dataset (50 simulations for each pair). We then counted the number of times a model fit the simulated data best (according to the LOOIC rule), effectively creating an $M \times M$ confusion matrix, where M is the number of models. In the case where we have a diagonal matrix of ones, the models are perfectly recoverable and hence as reliable as possible. See the model recovery results for the pooled sample in Table S5.

Table S5: Confusion matrix from the model recovery analysis of five RL models for a pooled dataset.

		**fit with**				
		M1	M2	M3	M4	M5
**generated**	M1	0.52	0.08	0.28	0.10	0.02
	M2	0.22	0.08	0.22	0.34	0.14
	M3	0.44	0.02	0.36	0.10	0.08
	M4	0.22	0.14	0.22	0.22	0.20
	M5	0.12	0.16	0.14	0.28	0.30

Cluster Id	Voxels	P	Z peak	X	Y	Z
2a	808	0.0015	4.28	36	-6	-6
2b			3.78	28	-4	-4
2c			3.72	16	4	2
2d			3.51	52	10	6
2e			3.47	24	12	-8
2f			3.23	50	-8	10
1a	468	0.0282	4.1	28	46	32
1b			3.51	14	18	34
1c			3.49	16	22	30
1d			3.39	16	32	32

Table S6: Activation clusters of increased activity in arthritis participants vs controls, associated with PPE and modulated by the punishment sensitivity parameter. Coordinates in MNI space.

Cluster Id	Voxels	P	Z peak	X	Y	Z
Arthritis > Controls						
1a	662	1.31e-06	3.76	-24	-82	-10
1b			3.75	-16	-82	-12
1c			3.21	-14	-88	-6
1d			3.11	-28	-74	-8
1e			2.89	-20	-64	-8
1f			2.81	-34	-78	-12
Arthritis < Controls						
1a	218	1.65	3.59	16	44	22
1b			2.72	8	50	14
1c			2.48	6	52	20

Table S7: Activation clusters of increased and decreased activity in arthritis patients vs controls, associated with RPE and modulated by the reward sensitivity parameter. Coordinates in MNI space.

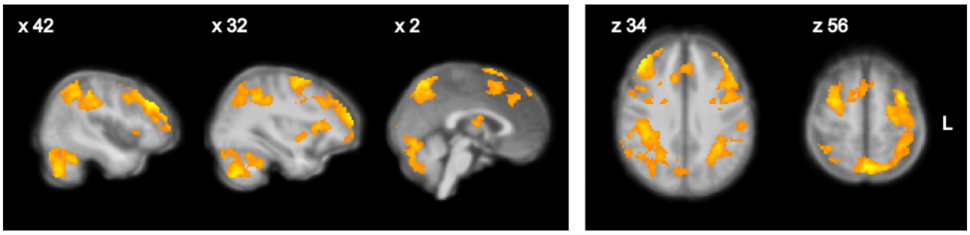

Figure S3: **Neural correlates of punishment prediction error shared across the arthritis and control groups.** Colorscale represents z scores ranging from 0 to 5, thresholded at z>2.6, p<0.05.

Cluster ID	Voxels	P	Z peak	X	Y	Z
2a	16180	5.48e-30	5.62	42	36	34
2b			5.34	-32	0	56
2c			4.97	2	-64	54
2d			4.91	-44	20	38
2e			4.83	4	-72	50
2f			4.83	-4	-66	56
1a	8416	3.53e-19	4.81	32	-70	-44
1b			4.76	-34	-48	-30
1c			4.59	42	-66	-38
1d			4.58	-40	-56	-44
1e			4.57	-26	-66	-40
1f			4.57	20	-78	-20

Table S8: Activation clusters associated with punishment prediction error, common activity across patient and control groups. Coordinates in MNI space.

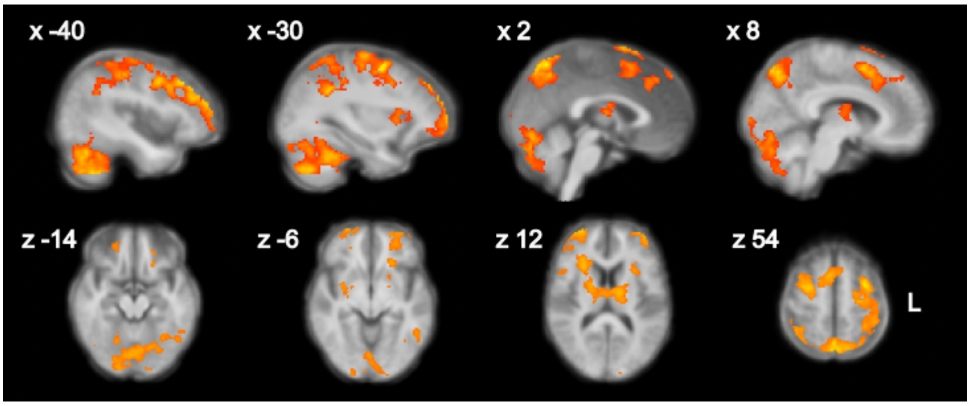

Figure S4: **Neural correlates of reward prediction error shared across the arthritis and control groups.** Colorscale represents z scores ranging from 0 to 5, thresholded at z>2.6, p<0.05.

]

Cluster ID	Voxels	P	Z peak	X	Y	Z
5a	34881	0	7.08	12	-86	-14
5b			6.59	-26	-76	-16
5c			6.52	26	-82	-16
5d			6.38	22	-84	-16
5e			6.35	-26	-84	-18
5f			6.22	26	-92	2
4a	2285	4.92E-17	4.47	2	-12	12
4b			3.83	8	-12	12
4c			3.79	8	-22	10
4d			3.79	28	4	24
4e			3.78	-10	-12	12
4f			3.72	28	10	18
3a	1381	9.49E-12	4.38	-30	2	54
3b			3.9	-38	0	24
3c			3.71	-40	4	24
3d			3.7	-42	4	34
3e			3.7	-38	28	24
3f			3.53	-38	30	38
2a	712	5.36E-07	3.81	-32	56	-6
2b			3.71	-20	52	-12
2c			3.54	-32	50	-8
2d			3.47	-28	42	-8
2e			3.46	-28	40	-12
2f			3.46	-26	60	6
1a	202	0.0346	3.98	10	56	-12
1b			3.94	12	58	-8
1c			3.6	8	60	-6
1d			3.35	26	60	-6
1e			3.09	26	56	-8
1f			2.92	20	52	-10

Table S9: Activation clusters associated with reward prediction error, positive mean activity across patient and control groups. Coordinates in MNI space.

Supplementary imaging results

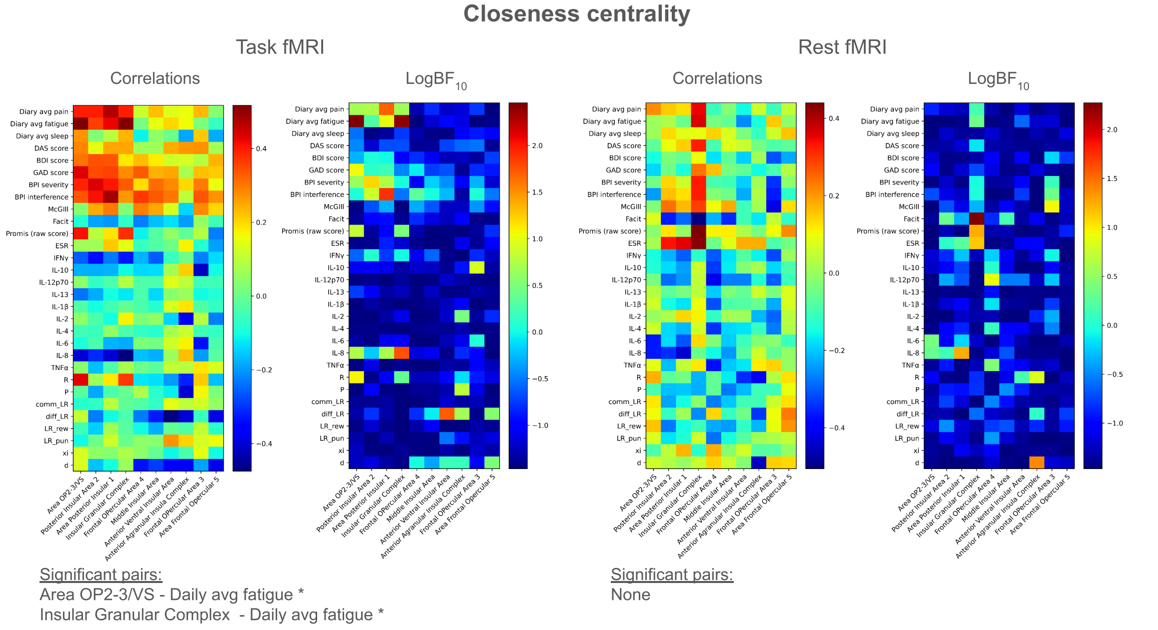

Figure S5: Closeness centrality correlation results. Please refer to the supplementary file with network analysis results for precise values of Pearson's R and LogBF10. * denotes BF>10 and ** denotes BF>30. The results are not FDR corrected and intended to be exploratory.

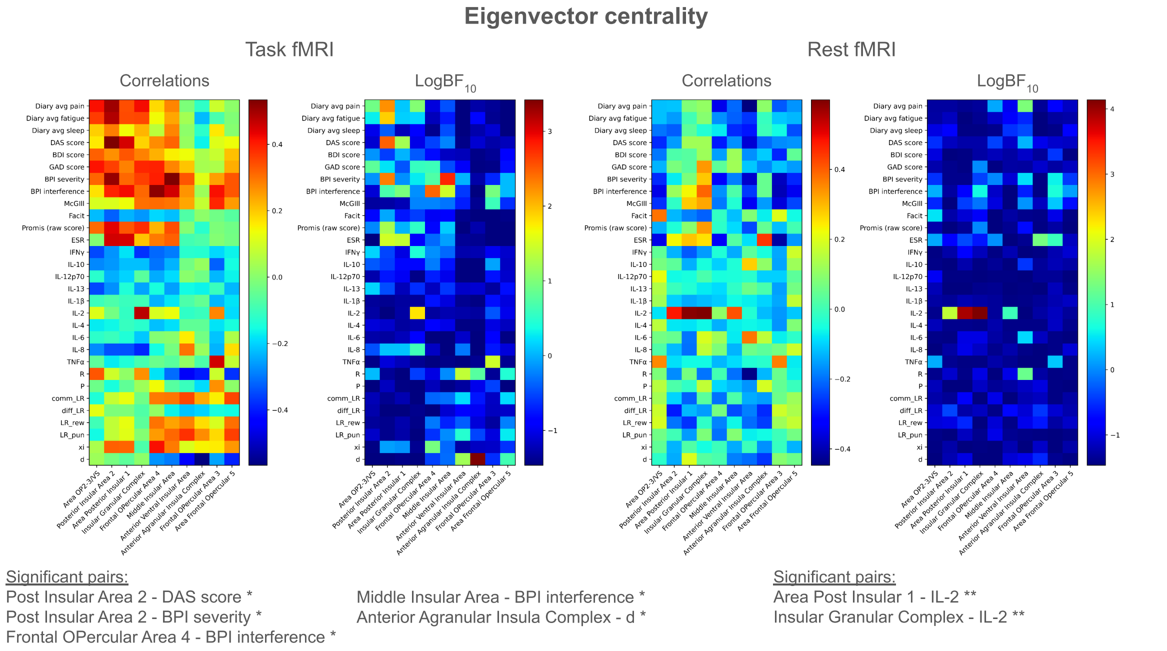

Figure S6: Eigenvector centrality correlation results. Please refer to the supplementary file with network analysis results for precise values of Pearson's R and LogBF10. * denotes BF>10 and ** denotes BF>30. The results are not FDR corrected and intended to be exploratory.

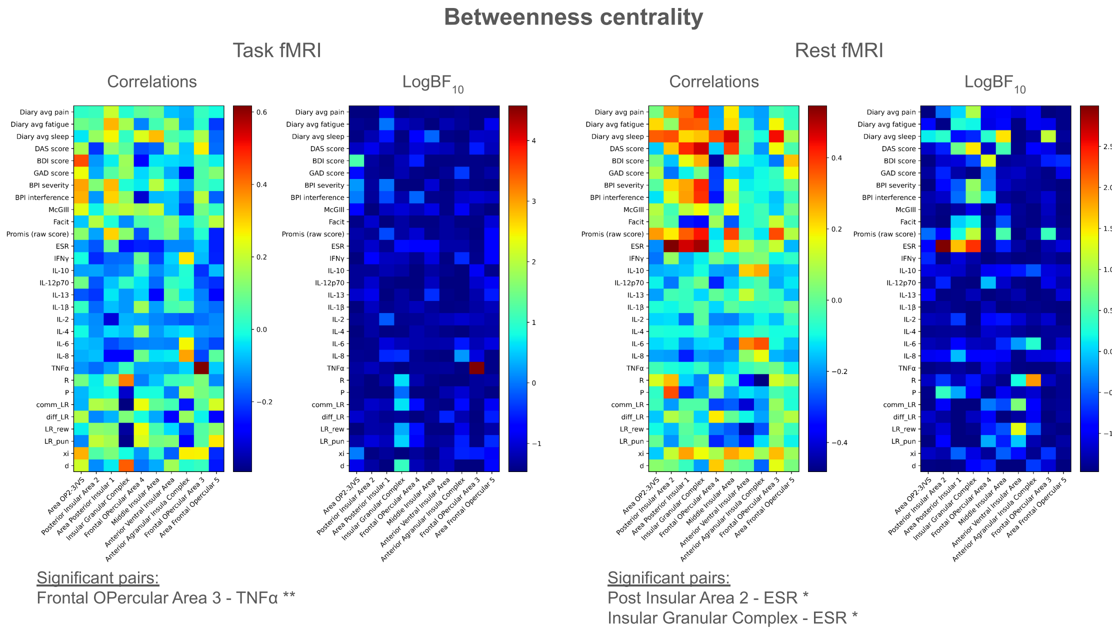

Figure S7: Betweenness centrality correlation results. Please refer to the supplementary file with network analysis results for precise values of Pearson's R and LogBF10. * denotes BF>10 and ** denotes BF>30. The results are not FDR corrected and intended to be exploratory.

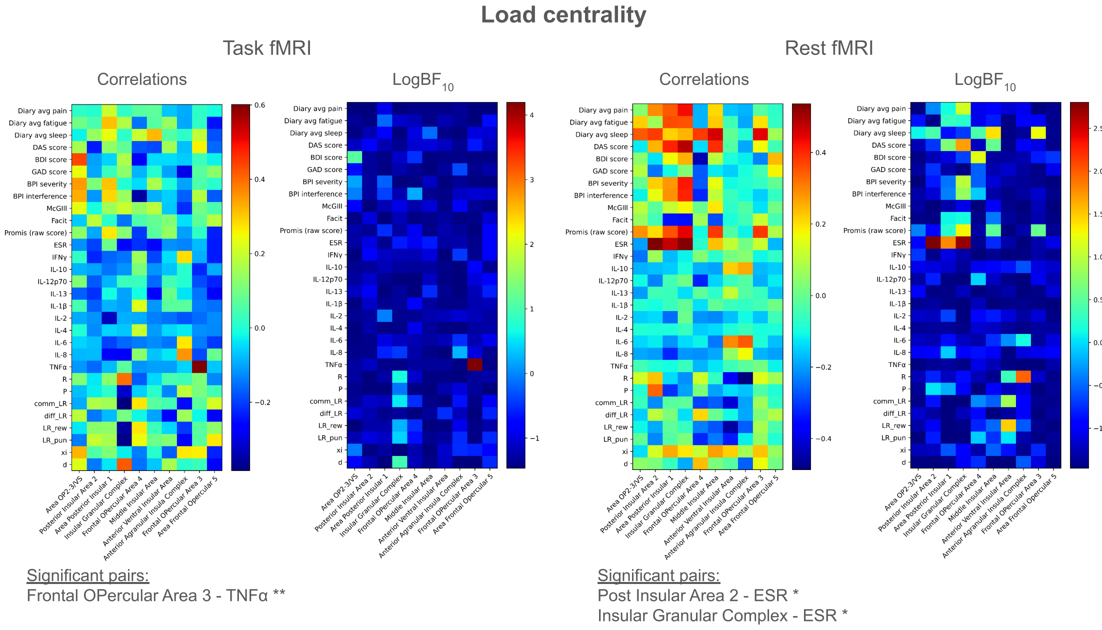

Figure S8: Load centrality correlation results. Please refer to the supplementary file with network analysis results for precise values of Pearson's R and LogBF10. * denotes BF>10 and ** denotes BF>30. The results are not FDR corrected and intended to be exploratory.
